# Potential influence of temperature and precipitation on preterm birth rate in Puerto Rico

**DOI:** 10.1038/s41598-018-34179-z

**Published:** 2018-10-31

**Authors:** Xue Yu, Zlatan Feric, José F. Cordero, John D. Meeker, Akram Alshawabkeh

**Affiliations:** 1 Department of Civil and Environmental Engineering, Northeastern University, Boston, MA USA; 2 Department of Epidemiology and Biostatistics, College of Public Health, University of Georgia, Athens, GA USA; 3 Department of Environmental Health Sciences, School of Public Health, University of Michigan, Ann Arbor, MI USA

## Abstract

The preterm birth (PTB) rate for singletons born in the tropical Caribbean island Puerto Rico increased from 11.3% in 1994, which was comparable to rates in the U.S., to as high as 18.3% in 2006 before decreasing to 15.5% in 2012. A few studies have reported that weather extremes are associated with higher risk of preterm birth, however, the effects of ambient temperature and precipitation has not been well examined in Puerto Rico. We compiled child birth data from the National Center for Health Statistics and weather data from the National Oceanic and Atmospheric Administration from 1994 to 2012. We explored the association between the weather factors and PTB rates with a distributed lag non-linear model (DLNM). We did not find direct association of lagged effect of temperature on birth outcome over monthly timescales. Both high intensity and frequency of precipitation and high frequency of storm and flood events are associated with increased risk of PTB rates. While the weather factors do not explain the marked increase and decrease in PTB rate, we emphasize the negative effects on PTB from weather extremes particularly precipitation in Puerto Rico.

## Introduction

Preterm birth (PTB), defined as birth before 37 weeks of completed gestation, is the leading cause of neonatal morbidity in the U.S., and is also associated with a number of chronic health conditions and developmental disabilities that cause lifelong consequences^1–5^. Despite its enormous social and economic cost, currently no model can precisely or roughly predict the occurrence of PTB.

In addition to changes in obstetric practices, multidimensional factors are found to contribute to PTB including personal characteristics such as genetics, behavioral and psychological factors, medical conditions, infertility treatment and biology, and environmental influences such as contaminant exposures and climate disturbances^6,7^. Many of the epigenetic factors are interactive and connected and are collectively reflected as the socioeconomic status of the individual, where individuals of disadvantaged socioeconomic status are linked with poorer medical conditions and infertility treatment, and more intense mental health problems. Despite consideration of socioeconomic variabilities, the ethnic disparities of PTB rates between white (~11%) and black (~16%) infants in the U.S. remains a puzzle given current knowledge^8,9^. In addition, many researchers have explored the effects of environmental contaminant exposure, and have identified several pollutants contributing to PTB, including air pollutants such as particulate matter and sulfur dioxide^10,11^, drinking water pollutants such as atrazine and nitrate^12^, and other man-made pollutants such as bisphenol^13^, phthalate^14^, and pesticides^15^. Studies that explore the effects of weather conditions on PTB rates generally conclude that higher temperatures increase the odds of PTB^16–23^; however, other studies have found a protective effect of higher temperature on PTB selected regions^24^ or no conclusive effect^25^. High temperature has both negative biological and psychological effect and hence is plausible to be associated with the increase in PTB rates^16,17^. On the other hand, no study has quantified the effect of precipitation on PTB, though heavy precipitation has been noticed to exacerbate the negative maternal and pregnancy outcomes^26^ and also storm and flood events are considered to be stresses during pregnancy^27^.

**Figure 1:**
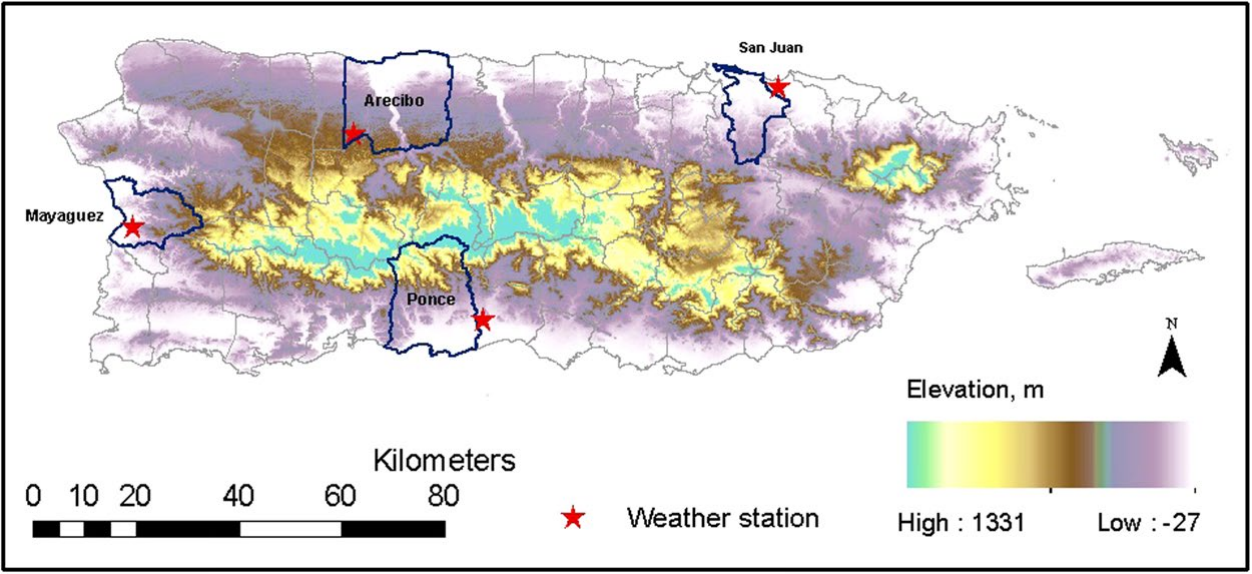
Locations of three selected municipals, i.e. Mayagüez, San Juan, and Ponce in Puerto Rico; and the three corresponding weather stations.

Ambient temperature and precipitation exert no immediate or direct effect on PTB, instead, the weather factors play as a trigger to the occurrence of adverse birth outcome^23^. Here we use comprehensive datasets of child natality and weather information for Puerto Rico to assess the integrative weather effects on PTB (Fig. 1). We hypothesize that both increased precipitation and higher temperature increase the odd of PTB in Puerto Rico.

## Results

**Figure 2:**
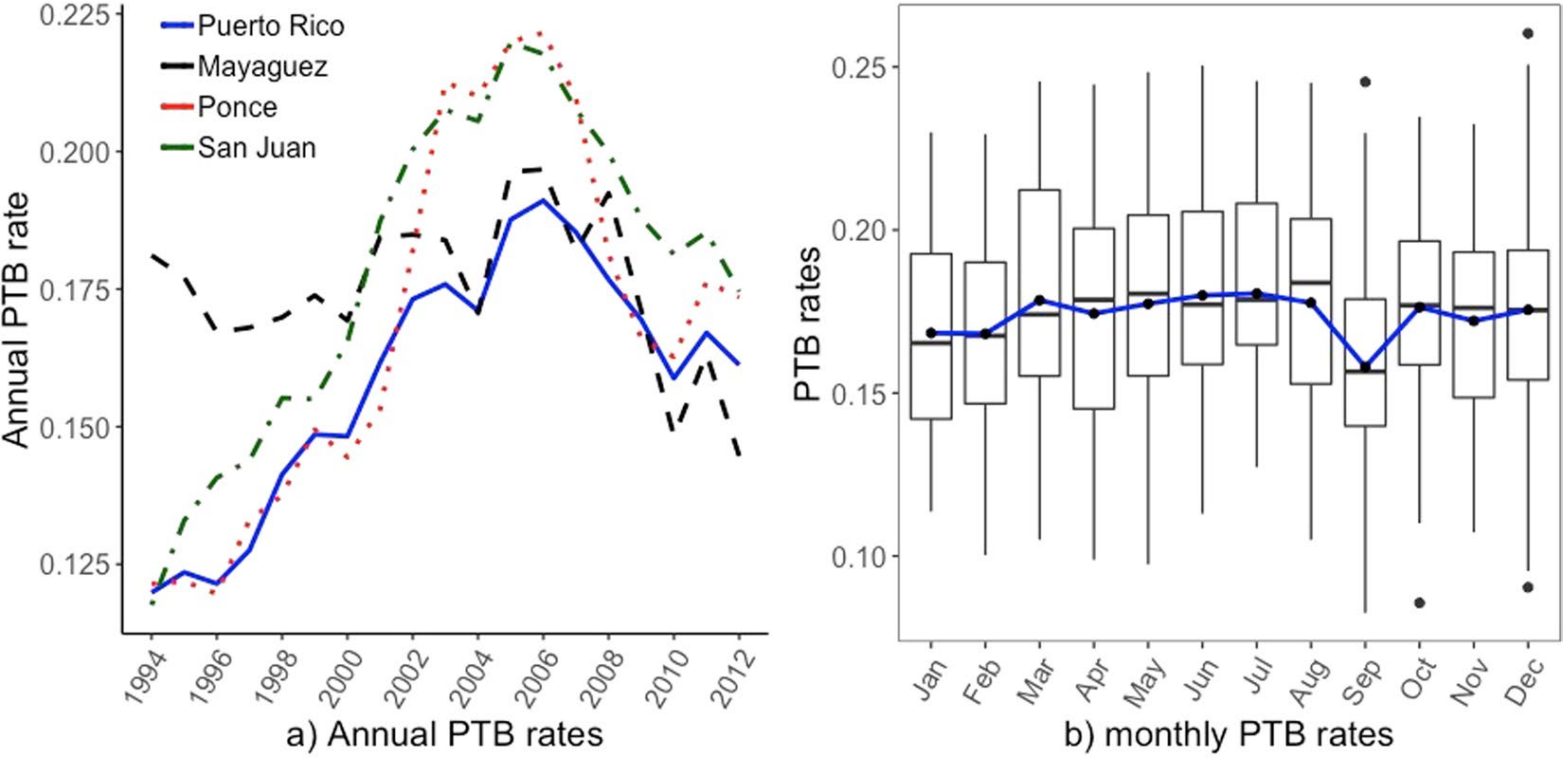
Annual distributions of PTB rates of single born babies from 1994 to 2012 (plot a) and monthly PTB rates (plot b) in Puerto Rico.

In Puerto Rico, the overall number of singleton born babies in the 19 years of this study is 1,005,340, where 14.5% of them are PTB. The total numbers of births and the associated PTB rates exhibit different temporal patterns. The total annual number of births decreases steadily from around 62,940 in 1994 to 39,200 in 2012 (Appendix Fig. A.1). On the other hand, the PTB rate for singleton babies increases from 11.3% in 1994 to 18.3% in 2006 then decreases to 15.5% in 2012, and 11.8% in 2014 (Fig. 2). The lowest PTB rate occurs in September (Fig. 2). The order of magnitudes of the numbers of child born and PTB rates are presented in Appendix Figs A.2–A.4.

PTB rates vary in different regions of Puerto Rico (Table 1), with the highest values in the metropolitan area of San Juan (16.3% out of the overall child birth: n = 213,720) and Mayagüez (16.4%, n = 71,390), followed by Ponce (15.4%, n = 118,820). The grouped areas of small metropolitans have an average PTB rate of 13.9% (n = 327,930), and the PTB rate in the non-metropolitan region is 12.2% (n = 9,760). The temporal patterns of PTB rates in Ponce, San Juan, and the whole island have anti U-shapes, while there is not remarkable cross year variation but large within year variation of PTB rates in Mayagüez (Fig. 2).

**Table 1:** Basic statistics of weather variables at the four stations in Puerto Rico.

Region	Variable	Mean	Median	Min	Max	Std
Mayagüez	rain	96.3	75.7	0	392	81.8
Ponce	rain	83.7	58.6	0	594.9	86.7
San Juan	rain	128.8	117.6	0	471.5	78.7
Mayagüez	DP10	1	1	0	7	1.3
Ponce	DP10	0.8	0	1	8	1.2
San Juan	DP10	1.1	1	0	6	1.2
Mayagüez	TAVG	26.1	26.1	23.1	29.3	1.5
Ponce	TAVG	26.6	26.8	23.1	28.9	1.3
San Juan	TAVG	27	27.2	24	29.8	1.4
Mayagüez	DX90	9.1	5	0	31	9.9
Ponce	DX90	17.3	19	0	31	10.6
San Juan	DX90	5.5	3	0	30	6.3
Mayagüez	storm	0.4	0	0	8	1.2
Ponce	storm	0.3	0	0	6	0.9
San Juan	storm	0.8	0	0	8	1.7
Mayagüez	flood	0.6	0	0	6	1.2
Ponce	flood	0.4	0	0	8	1.2
San Juan	flood	1.1	0	0	8	1.7
Mayagüez	PTB	17.5	17.5	8.6	25.1	2.8
Ponce	PTB	16.8	16.5	8.3	26.0	3.9
San Juan	PTB	17.8	18.1	10.0	24.6	3.3
Puerto Rico	PTB	15.8	16.2	10.6	20.8	2.4

Note: rain: monthly rain intensity (mm); DP10: number of days

with >=25.4 mm rain in the month; TAVG: monthly average

temperature; DX90: number of days with >=32.2 °C in the month;

storm and flood: count of events in a month. Min, Max and Std

stand for minimum, maximum and standard deviation, respectively.

**Figure 3:**
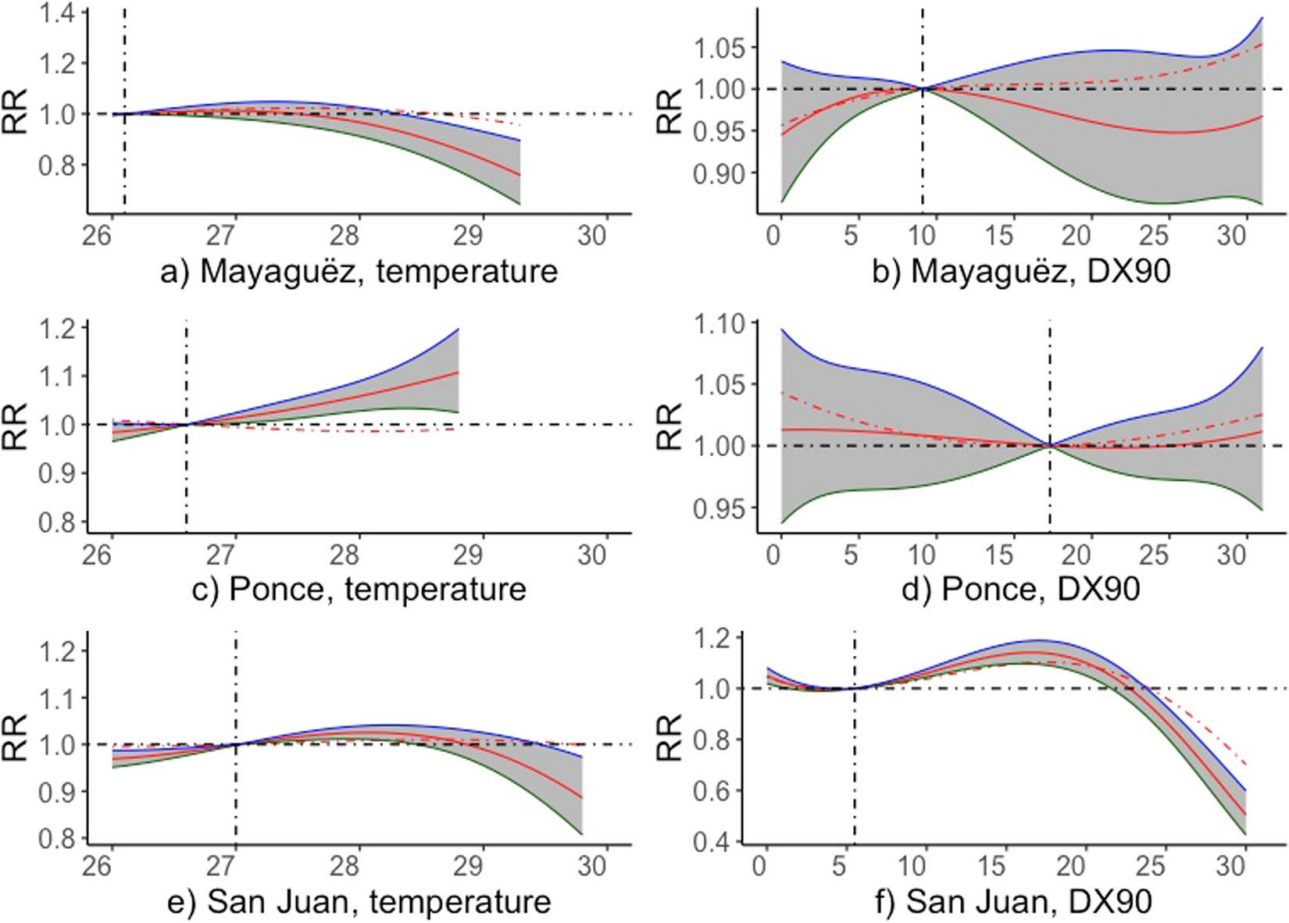
Cumulative relative risk (RR) of PTB rate in respect to the mean values for average temperature (left panels) and the number of days in a month with temperature above 32 °C (DX90) subjected to time lags up to 1 months in Mayagüez, Ponce and San Juan. The horizontal broken line represents the reference line, i.e. RR = 1. The red broken and solid lines represent 0- and 1- month lags, respectively. The blue and dark-green lines along the shaded area represent 95% confidence interval values. The vertical broken lines represent mean values. The line and color notations are consistent for all the plots.

The effects of temperature on PTB are presented in Fig. 3. At both areas of Mayagüez and San Juan, temperature shows little or protective effects to PTB, while in Ponce, temperature slightly increases the RR of PTB. At both Mayagüez and Ponce, the 1-month lagged effects are smaller than that of no lag effect and show little or protect effect on PTB. At San Juan, there is little difference between the 1-month lagged effect and no lag effect of warmer months on PTB.

**Figure 4:**
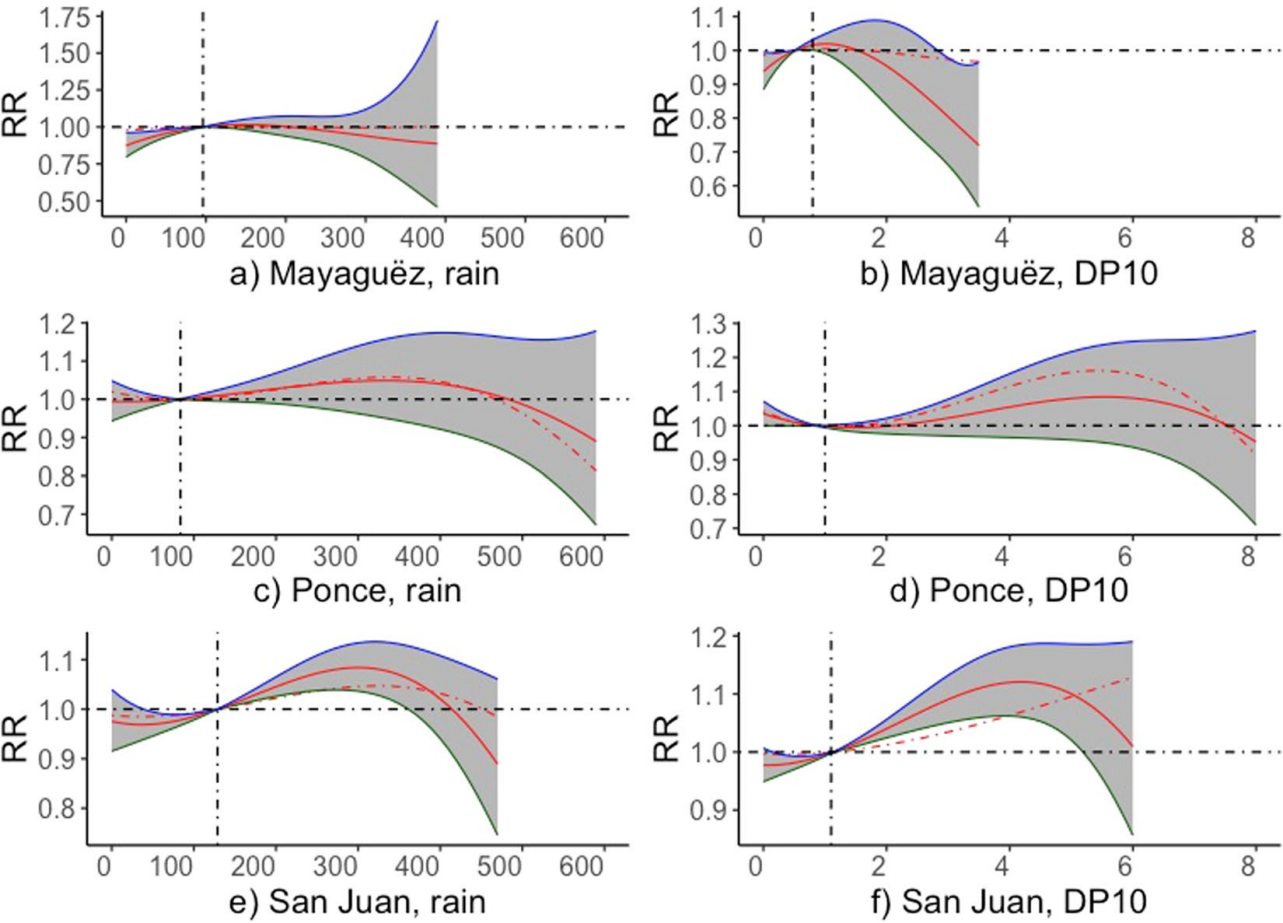
Cumulative RR of PTB rates in respect to the mean values for monthly rain intensities (mm) and frequencies (DP10, number of days with rain intensities above 254 mm in a month) subjected to time lags up to 1 months in municipals of Puerto Rico.

We present the overall effect of precipitation intensity and frequency on PTB rates for up to 1-month lag for the three regions of Puerto Rico (Fig. 4). At both Mayagüez and Ponce, the 1-month lagged effects from precipitation (both intensity and frequency) are smaller than non-lagged precipitation on PTB, while at Ponce the lagged effects are greater than the non-lagged effects. Generally, higher precipitation both in intensity and frequency show some increased effects on PTB in Puerto Rico.

**Figure 5:**
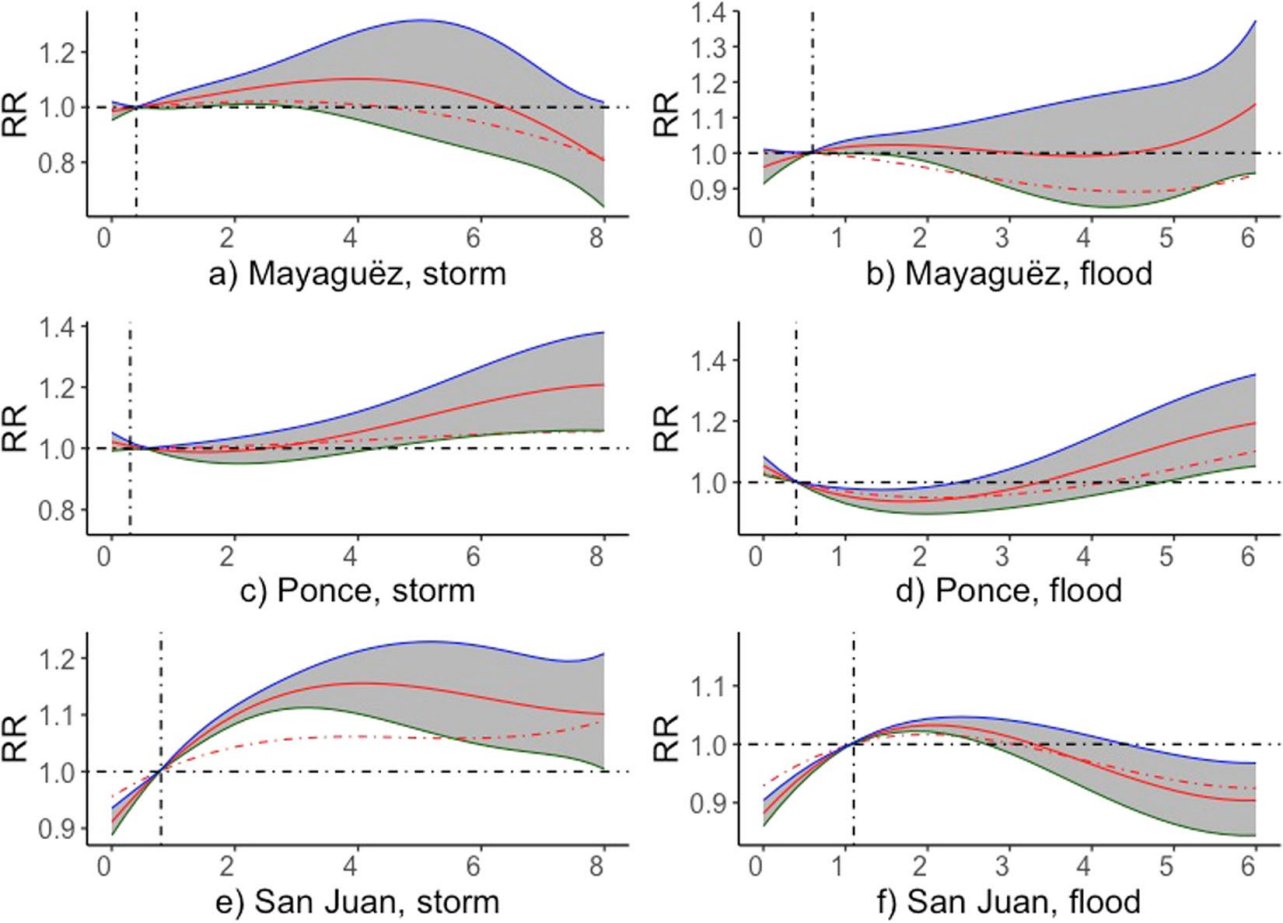
Cumulative RR of PTB rates in respect to the mean values for monthly frequencies of storms and flood subjected to time lags up to 1 months in municipals of Puerto Rico.

Our results show that high frequencies of storm and flood events increase the risk of PTB rates considerably for mothers of most demographic profiles in the three regions (Fig. 5). Moreover, the 1-month lagged storm events show increased effect on the risk of PTB rates in all three regions.

## Discussion

We observed mixed effects of temperature on PTB rates for different regions, though generally temperature appears to be a protective factor or has no direct effect on preterm birth for this tropical island. In comparison, Cox *et al*.^22^ reports RR values increase from 1.02 to 1.15 when the daily minimum temperature increases from 8.3 to 16.3 °C and of 1.05 to 1.28 when the daily maximum temperature increases from 14.7 to 26.5 °C on a 1-day lag basis for a temperate climate environment in Belgium, Europe. In the same literature, Cox *et al*.^22^ summarizes studies of temperature-related increase in PTB rates for both temperate and warmer climates and find inconclusive results. Zhang *et al*.^23^ also notes the mixed effects of temperature on PTB through a meta-analysis on existing literature, and they conclude that the current knowledge suggests that heat has stronger adverse effect than cold. The previous literature that report positive association between temperature and PTB rates propose various values of RR, such as a 8.6% increase for a 5.6 °C temperature before delivery in California, U.S.^28^, a 1.87% increase for 10 °C in Rome, Italy^20^, and a 5–20% increase of a 2 days lag in Valencia, Spain^29^. He *et al*.^30^ finds both a positive association between extreme cold (17.9% for the 1^st^ percentile temperature of 7.6 °C) and extreme hot (10% for 99^th^ percentile of 31.9 °C) temperatures and PTB rates in Guangzhou, China. Comparatively several studies report protective effects of temperature on PTB rates, where high temperature is a protective factor^24,31^. Liang *et al*.^24^ reports that low temperatures (the 1^st^ percentile temperature of 9 °C) have a RR on PTB rates of 1.54, while high temperatures have a RR of 0.62 (99^th^ percentile) for 30 days before delivery in Shenzhen, China.

Our approach to analyzing the association between temperature and PTB rate is different from the literature, as we use both the frequency of high temperature days in a month and the actual temperature values, due to the temperature patterns in Puerto Rico. There is no considerable variation of temperature from year to year in Puerto Rico (Appendix Figs A.2–A.4), but there exists noticeable yet small seasonal variations (Fig. 2). We found no lagged effect of temperature on PTB rates over monthly timescales. Exposure to high temperature is linked to increased blood flow^32^, dehydration^33^, pre-eclampsia^34^, and other negative effects on maternal health and birth outcome. The effect of temperature on birth outcome is based on acute temporal association on the day/week of birth. The biological mechanisms of thermoregulation that are happening acutely during heat stress induce contractions and could lead to preterm labor and ultimately preterm birth^32–34^. This acute heat stress effect of temperature on PTB might explain that in this study temperature somewhat exhibits as a protective effect on PTB because there is only mild variation of temperature in Puerto Rico.

We studied the association between precipitation and PTB rates, which is largely overlooked by literature. Lee *et al*.^35^ reports that PTB rates are 3 times higher in the dry seasons than in the rainy seasons for the country of Zimbabwe; however, this might be more attributed to the effects of high temperature and the poor availability of drinking water. Both precipitation intensity and frequency of extremes appear to be a larger factor than the frequency of temperature extremes in association with the risk of PTB rates in our study. We clearly observe increased effects on PTB rates from both extreme levels of precipitation intensity and monthly frequency of high precipitation days, and in contrast the mean levels of precipitation intensity or frequency (95% confidence intervals) have little effect on PTB rates. This phenomenon points out that rather than the actual values of precipitation or temperature, it is the extreme climate events that have more impact on PTB, which is consistent with findings from literature^36^. Our findings highlight the concern for increased risk of adverse birth outcomes under the global warming scenario, which likely trigger more intense and frequent precipitation as suggested by both simulation and observation results^37,38^.

Our study found that frequencies of storm and flood events are highly associated with increased risk of PTB rates. Recent evidence suggests that stress in pregnancy can have adverse effects on birth outcomes^39^. Maternal care might be disrupted during the storm events in addition to stress. However, on the other hand the pregnant women may be more careful about their health and be better taken care of by their family members. Hence the overall effect from storm events on PTB are subtle and sensitive to specification of the pregnant women’s socio-economic status^27^. Several studies, including the present study, highlight the trends over time in PTB rates climbing through the 1990s up until the mid-2000s, then gradually decreasing. This overall pattern in the U.S. has been previously attributed to several factors unrelated to weather, including obstetrics/gynecology standards practices around cesarean section delivery^2^ (Appendix Fig. A.5). In this study weather effects, especially precipitation and storm are associated with increased risk of PTB rates. Mothers in Puerto Rico may be more sensitive to storm and flood events and hence the temporal occurrences of storm and flood events may put stress on mothers and may somewhat influence the temporal trend of PTB rates in Puerto Rico.

The mechanism of how precipitation affects birth is unknown, though several studies point out that high precipitation is negatively related to human health. There are many intermediate factors associated with precipitation that impact PTB, such as environmental contamination and disease transmission that are not directly explored in this study. Although no study has quantitatively explored the association between precipitation and PTB, a comparative study on human health found that precipitation extremes are associated with changes to air moisture content^40^, which affects respiratory systems and increases the risk of asthma^41^. Heavy rain events facilitate the transmission of waterborne disease such as diarrhea, dengue fever, Chikungunya, and Zika viruses^42–45^. Indeed, Johansson *et al*.^46^ studied the local and global effects of climate on dengue transmission in Puerto Rico, which increased from 10,508 cases in 2007 to 26,766 cases in 2010, and confirmed the importance of temperature and precipitation in the transmission of dengue on this island. The mosquito-borne dengue, Chikungunya, and Zika viruses have already spread to Puerto Rico as of 2016 and can be transmitted more efficiently with high intensities and frequencies of precipitation events^47^. Zika virus is harmful to pregnant women and fetuses, and increases the rate of microcephaly in newborns in particular^48^. The northern coast of Puerto Rico contains many karst aquifers, which serve as a significant source of the total water production for this island^49^. Precipitation is the main recharge to the karst aquifers, where rainfall passes through the limestone outcrop rapidly through the complex karst conduit network, fractures and sinkholes, and the quick flush of both surface water and groundwater makes the karst rather vulnerable to contamination^50^. Precipitation intensity and frequency extremes have been linked to the increase of the concentrations of chemical contaminants in soil and groundwater^51^. Moreover, this area has one of the highest densities of Superfund, and Resource Conservation and Recovery Act - Corrective Action (RCRA-CA) sites in the U.S.^52^. In a synoptic survey study, Cantonwine *et al*.^53^ found widespread exposure to phthalates among pregnant women living in this karst area particularly for those groups using private wells as drinking water, which strongly indicates the association of exposure to environmental chemicals and incidences of PTB in Puerto Rico. The increase and decrease trend of PTB rates that roughly coincide with the historical contaminants accumulation and recent reduction due to remediation procedures is a further evidence to support this link.

Our analysis should be considered in light of limitations. Numerous studies using daily data have found significant positive associations between heat waves, with 0 to 7- day lags, and PTB in the last month of gestation^21,54,55^. Heat waves are typically defined by extreme temperatures (e.g. 95^th^ percentile for summer time daily average temperatures or maximum temperatures) for at least 2 days. This relationship is likely due to increased risk of premature labor and/or premature rupture of membranes following heat stress and/or dehydration^7^. We used month as the lowest time scale for the following reasons: (1) only monthly level child birth natality data is publicly available from CDC; (2) Puerto Rico has general stable warm temperatures and monthly average temperature can capture the temperature variance sufficiently; and (3) the etiology threshold window for temperature effect on preterm birth is still debating and 1-monthly exposure before delivery is plausible to explore the short term effect of temperature exposure^56^. Nevertheless, this study might underestimate the near-birth association between temperature and PTB, as most prior studies examined their association at a daily level. Moreover, the effects of meteorological factors on health might be modified at different time lags in the short-term scale, for example, a study found that relative humidity showed negative effect at lag 1 day and positive effect at lag 5–7 days on adolescent hand, foot, and mouth disease^57^. Moreover, there are many more important factors such as gene, environmental pollution, social-economy status and health care affect PTB rates, whereas the effects of extreme weather conditions exhibit more as a “trigger” to negative birth outcomes^58^. In recent decades, the total number of child births dropped significantly but the drop in PTB rate has not followed the same decreasing rate. Reasons for the changes to PTB rates in Puerto Rico may not be clearly understood, and maybe influenced by the change in obstetric practices, recent economic downslide and population migration trend^59^, but the contributions of weather-intensified virus epidemics as well as exposure to environmental contaminants cannot be neglected.

## Methods

We compiled and analyzed 19 years (1994–2012) of child birth certificates data from the National Center for Health Statistics in Centers for Disease Control and Prevention (NCHS CDC) representing the most comprehensive knowledge of maternal and child birth information in Puerto Rico^60^. This natality data set includes information on mother’s residence, mother’s characteristics, birth characteristics, and maternal risk factors. The lowest level of geographic information is identified as areas with more than 100,000 people, and in Puerto Rico we have Bayamón, Caguas, Carolina, Mayagüez, Ponce, San Juan, nonmetropolitan areas, and combined areas with population less than 10,000. For representative spatial analysis, data completeness and geographic match of weather stations, in this study we chose three regions including San Juan, Mayagüez, and Ponce which represents 44% of the total child birth records (458,820 out of 1,044,640) in the study period (Appendix Fig. A.1). San Juan is the capital of Puerto Rico and is the most developed and populated area representing a metropolitan setting. Mayagüez is in the western part of the island and is a medium-sized city with the most varied climate of Puerto Rico. Ponce is the most populous city outside of the San Juan metropolitan area and is located in southern Puerto Rico and is the warmest among the three representative regions. The lowest level of time information on child birth from this data set is month in a year. The gestational information is documented as the number of weeks completed. PTB rate is calculated as the number of child birth with completed gestation weeks less than 37 to the total number of child birth in each time span (month or year). We only explored singleton data while ignoring twin, triplet or more birth data for homogenous analysis.

We compiled the monthly weather data from the National Oceanic and Atmospheric Administration (NOAA) in the same period at three selected stations where the majority of the population reside to represent weather conditions in the corresponding locations^61^. The three weather stations are: Luis Muñoz Marín International Airport in San Juan (Station ID: RQW00011641; elevation: 2.7 m above sea level), Mayagüez City Station (ID: RQC00666073; elevation: 22.6 m), and Ponce 4 E station (ID: RQC00667292; elevation: 21.3 m) (Fig. 1). We matched each birth record location identifier to its closest weather station. We used NOAA suggested critical values of 254 mm (variable: DP10) per day for extreme precipitation and 32 °C (variable: DX90) for extreme daily temperature in our study as convenience, which represent 90% and 80% percentiles of their entire records respectively. The frequency of daily precipitation intensity greater than 254 mm in a month suggests how wet the month is and is used as the variable frequency of heavy rain days, and the frequency of daily average temperature above 32 °C in a month suggests how warm the month is and is used as the variable frequency of high temperature days. Meanwhile we collected storm and flood information from NOAA^56^. We defined the events of “heavy rain”,”hurricane”,”tornado”, and “tropical storm” as storm events, and both the events of “flash flood” and “flood” as flood from this data set.

The temporal patterns of the weather factors in the three regions can be found in Appendix Figs A.2–A.4. The general information of the weather factors can be found in Table 1. Of the 228 months in our study period, 90 or 40% of the months contain no single extreme wet day with more than 254 mm, and only 1 month has 6 extremely wet days. The monthly average temperature in San Juan has a mean of 27.0 °C (95% CI: 26.8–27.2 °C). Of the total 228 months, 64 or 28% of them have no single day warmer than 32 °C, and only 17 months (7%) have more than 15 high temperature days. Since there is not much variation in the actual monthly temperature values (Fig. 2), the frequency of high temperature days in a month is used to evaluate the temperature effect on PTB. Puerto Rico experiences more frequent storms especially after 2003.

We first performed a general exploration of the data, including descriptive statistics, Pearson correlation, time series examination, and ANOVA for group means comparison. We then performed a logistic regression analysis and panel linear model (plm) to examine the modeling ability by using the weather factors to predict the occurrence of PTB. Based on both the logistic regression and plm analyses, we did not obtain any convincing results to build a strong association between weather factors and PTB rate (Appendix 1). This lack of direct relationship has been noted by previous studies and indicates that the relationship is non-linear^10,24^. We used the distributed lag non-linear model (DLNM) to capture and explore the delayed effects of weather factors on PTB rates. The essence of the DLNM model is to apply a generalized linear model by fitting a cross-basis (*cb*) function of the independent variable^62^. The cross-basis function pictures both the shape and the distributed lagged effects of the variable (more details are available in literature)^62,63^. The general form of the DLNM model is:

$$PTB\_count=cb({x}_{1})+total\_birth+month$$

where x_1_ is the weather factor and month is from 1 to 12 in every year. We used Poisson distribution in our model. Our data showed that there is little variation in RMSE and AIC values for the changes of degrees for the polynomial fitting and the number of lags, though there are small variations in the shape of the model outputs. Our final model selection choice was to assign 1-month lag and 3-degrees of polynomial to all the weather factors. The effect of each weather factor on PTB was examined separately; therefore, x_1_ in the equation only refers to one of the factors exclusively, including precipitation intensity and frequency (DP10), temperature and warm temperature frequency (DX90), and frequencies of storm and flood events. The month variable is used to smooth the seasonal impact. We evaluated the lagged effects with relative risk (RR), which is calculated as the effect of a level of a weather factor on PTB against the effect from the mean value of that factor.

## Electronic supplementary material

Supplementary File

## Data Availability

Availability of the data sets used in the study is fully described in the Method section.
