# Supplementary material for: Potential influence of temperature and precipitation on preterm birth rate in Puerto Rico
Source: Sci Rep. 2018 Oct 31;8:16106. doi: 10.1038/s41598-018-34179-z (PMC6208375; doi:10.1038/s41598-018-34179-z)
Supplement: Supplementary file 1 — Supplementary File [file 41598_2018_34179_MOESM1_ESM.docx]

**Appendices**

**Potential influence of temperature and precipitation on preterm birth rate in Puerto Rico**

Xue Yu, Zlatan Feric, José F. Cordero, John D Meeker, and Akram Alshawabkeh

**Table of Contents**

**Appendix 1: Regression, including logistic regression and panel linear model.**

**Figures:**

**Climate conditions in San Juan, Mayagüez, and Ponce.**

**Fig. A.1.** Temporal pattern of annual child birth frequency in Puerto Rico, and three studies regions including Mayagüez, Ponce and San Juan.

**Fig. A.2.** Time series of weather factors in San Juan, Puerto Rico, including monthly precipitation intensity (PRCP, unit: mm), number of days in a month with precipitation above 254 mm to represent precipitation frequency (rain frequency, DP10), monthly average temperature (unit: ^°^C, TAVG), number of days in a month with temperature above 32 ^°^C to represent high temperature frequency (DX90), frequencies of storm and flood event days in a month. The blue line in the plot is the linear trend line. The notations in Fig. A. 1 – Fig. A.3 are the same.

**Fig. A.3.** Time series of weather factors in Mayagüez, west of Puerto Rico.

**Fig. A.4.** Time series of climatic factors in Ponce, south of Puerto Rico.

**Temporal patterns of total annual child birth frequency in Puerto Rico**

**Fig. A.5.** Correlation matrix of preterm birth rate (preterm), and associated socio-demographic factors such as rate of cesarean delivery (cesarean), rate of pregnant women with at least one year’s college education (education), rate of mother’s race as black (mrace), rate of interracial marriage (interrace) and rate of cigarette use during pregnancy (cigarette).

**Appendix 1. Regressions**

**Logistic regression**

We performed logistic regression to analyze the power of weather factors to predict PTB. We build the model as:

$$ptb=PRCP+DP01+DP05+DP10+DX70+DX90+TAVG+TMAX+TMIN+flood+strom+EMNT+EMXP+EMXT$$

Where the variables are:

**ptb:** 0 for full term birth, 1 for PTB;

**PRCP:** total annual precipitation, mm;

**DP01:** number of days with >= 0.01 inch in the year;

**DP10:** number of days with >= 0.1 inch in the year;

**DX70:** number of days with maximum temperature >= 70 degrees Fahrenheit/ 21.1 degrees Celsius;

**DX90:** number of days with maximum temperature >= 90 degrees Fahrenheit/ 32.2 degrees Celsius;

**TAVG:** Average annual temperature;

**TMAX:** average annual maximum temperature;

**TMIN:** average annual minimum temperature;

**Flood:** annual flood event;

**Storm:** annual storm event;

**EMNT:** Extreme minimum temperature for the year;

**EMXP:** Highest daily total of precipitation in the year;

**EMXT:** Extreme maximum temperature for the year.

We randomly split our data into training and testing datasets with a ration of 8:2. The logistic regression results for the training dataset are presented in Appendix Table 1. We found most of the weather factors are significant to predict PTB. However, the equivalent McFadden R^2^ is 0.02 which is very small. Using ROC curve method to evaluate the predictive accuracy of the logistic model, the area under the curve (AUC) is 0.52, which is close to 0.5 and is regarded poor in predicting.

Table 1 Logistic regression results

|  | Estimate | Std..Error | z.value | p-value | significance |
| --- | --- | --- | --- | --- | --- |
| (Intercept) | -1.51 | 0.19 | -7.88 | 0 | *** |
| PRCP | 0 | 0 | 3.6 | 0 | *** |
| DP01 | -0.01 | 0 | -4.72 | 0 | *** |
| DP05 | 0 | 0 | -0.21 | 0.84 |  |
| DP10 | -0.01 | 0.01 | -1.51 | 0.13 |  |
| DX70 | 0.02 | 0 | 4.67 | 0 | *** |
| DX90 | 0 | 0 | 2.88 | 0 | ** |
| TAVG | -0.33 | 0.1 | -3.26 | 0 | ** |
| TMAX | 0.15 | 0.05 | 2.9 | 0 | ** |
| TMIN | 0.2 | 0.05 | 4.05 | 0 | *** |
| flood | 0.02 | 0 | 6.66 | 0 | *** |
| storm | 0.04 | 0 | 14.2 | 0 | *** |
| EMNT | 0 | 0 | -0.68 | 0.5 |  |
| EMXP | 0 | 0 | -6.76 | 0 | *** |
| EMXT | -0.03 | 0.01 | -6.13 | 0 | *** |

**Panel linear model**

We further used Panel linear model to assess weather effects on PTB. Our model is:

$$ptb_{count}=Total_{birth}+DP01+DP05+DP10+DX70+DX90+TAVG+TMAX+TMIN+flood+strom+month$$

We used the R package ‘plm’ to perform our calculation, where the index is set as different locations, i.e. San Juan, Mayagüez and Ponce. We used fixed effect model to account for the differences in different locations. The significant factors from the fixed effect model is DX70 (p-value = 0.02) and storm (p-value = 1.2e-05). The R^2^ is 0.21.

Both the logistic regression and the plm analyses identified several significant factors relating to PTB. However, none of these models are powerful enough in predicting the occurrences of PTB.


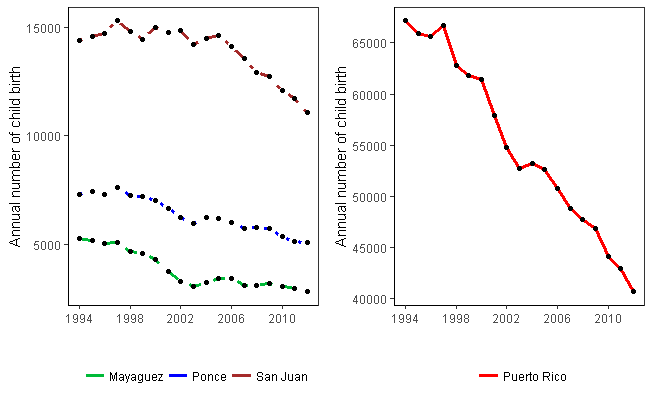


**Fig. A.1.** Temporal pattern of annual child birth frequency in Puerto Rico, and three studies regions including Mayagüez, Ponce and San Juan.


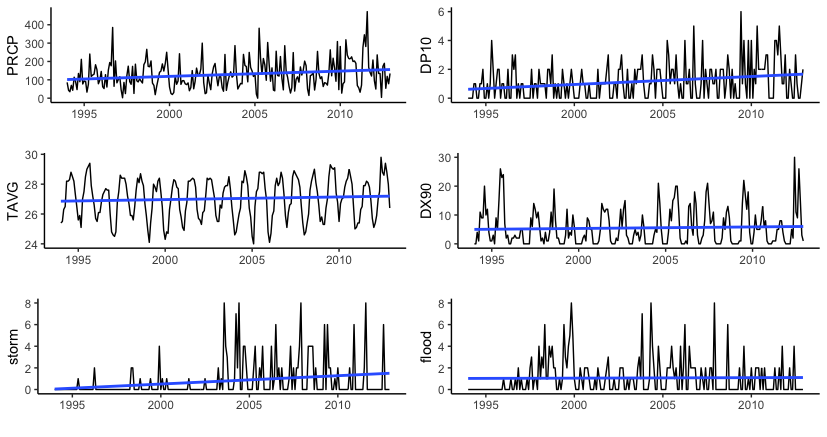


**Fig. A.2.** Time series of weather factors in San Juan, Puerto Rico, including monthly precipitation intensity (PRCP, unit: mm), number of days in a month with precipitation above 254 mm to represent precipitation frequency (rain frequency, DP10), monthly average temperature (unit: ^°^C, TAVG), number of days in a month with temperature above 32 ^°^C to represent high temperature frequency (DX90), frequencies of storm and flood event days in a month. The blue line in the plot is the linear trend line. The notations in Fig. A. 1 – Fig. A.3 are the same.


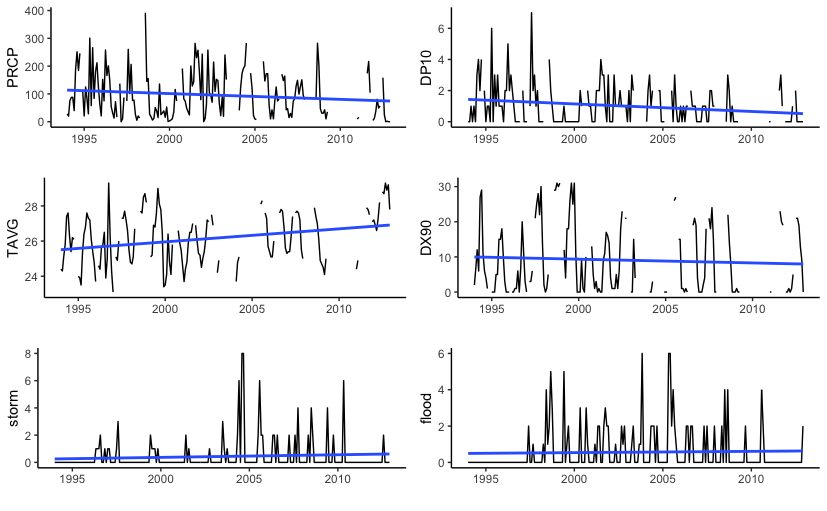


**Fig. A.3.** Time series of weather factors in Mayagüez, west of Puerto Rico.


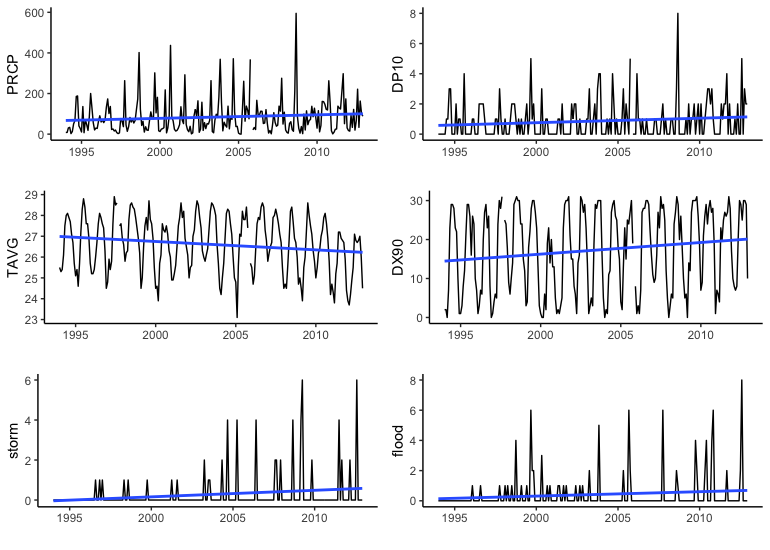


**Fig. A.4.** Time series of weather factors in Ponce, south of Puerto Rico.


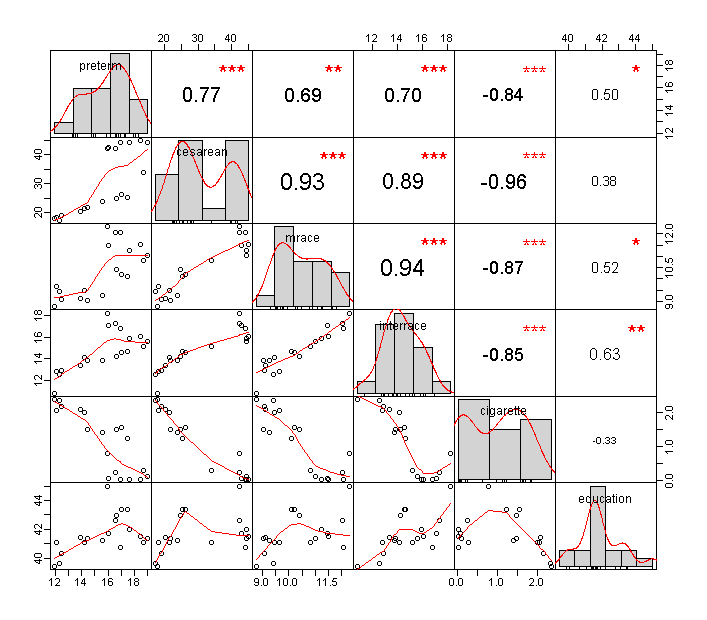


**Fig. A.5.** Correlation matrix of preterm birth rate (preterm), and associated socio-demographic factors such as rate of cesarean delivery (cesarean), rate of pregnant women with at least one year’s college education (education), rate of mother’s race as black (mrace), rate of interracial marriage (interrace) and rate of cigarette use during pregnancy (cigarette).
